# The Upregulation of COX2 in Human Degenerated Nucleus Pulposus: The Association of Inflammation with Intervertebral Disc Degeneration

**DOI:** 10.1155/2021/2933199

**Published:** 2021-10-18

**Authors:** Chang Liu, Guoyan Liang, Zhantao Deng, Jing Tan, Qiujian Zheng, Feng-Juan Lyu

**Affiliations:** ^1^Joint Center of South China University of Technology-The University of Western Australia for Regenerative Medicine Research, School of Medicine, South China University of Technology, Guangzhou 510006, China; ^2^Department of Orthopedics, Guangdong Provincial People's Hospital, Guangdong Academy of Medical Sciences, Guangzhou 510080, China

## Abstract

Intervertebral disc degeneration (IVDD) is an important risk factor of low back pain. We previously found upregulated markers of fibrosis, the late stage of chronic inflammation, in degenerated IVD with a small number of clinical specimens. Here, we aimed to study on a larger scale the association of cyclooxygenase 2 (COX2), an inflammation and/or pain marker, with IVDD. This study involved 107 LBP participants. The IVD degeneration level was graded on a 1–5 scale according to the Pfirrmann classification system. Discs at grades 1-3 were further grouped as white discs with grades 4-5 as black discs. We recorded baseline information about age, gender, body mass index (BMI), diabetes history, smoking history, and magnetic resonance imaging (MRI). Their association with IVDD was statistically analyzed. The expression level of COX2 was investigated by immunohistochemistry. The total integrated COX2 optical density (IOD), number of COX2-positive cells, and total cell number of each image were counted and analyzed by Image-Pro Plus software. The IOD and number of COX2-positive cells were divided by the total cell number to obtain COX2 expression density (IOD/cell) and COX2 positivity (cell+/cell). As a result, among the baseline information investigated, only age was found to have a significant association with IVDD. The IOD/cell was found to be significantly increased from grade 2 to grade 5, as well as in black discs compared to white discs. The cell+/cell displayed the same trend that it increased in highly degenerative discs compared to their counterparts. In conclusion, the expression of COX2 is associated with IVDD, which highlights COX2 as a biomarker for IVD degeneration and indicates the involvement of inflammation and pain signaling in IVDD.

## 1. Introduction

Low back pain (LBP) imposes huge social and economic burdens [1, 2]. It is estimated that about 80% of the world's population suffer from low back pain at least once in their lifetime. In the United States, LBP is the fifth leading cause of patient visits and the third leading cause of surgery [3]. LBP caused by internal disc disruption is defined as discogenic low back pain and is an important cause of LBP, accounting for about 42% of LBP [4]. Imaging examination shows that patients with low back pain are often accompanied by intervertebral disc degeneration (IVDD) [5, 6].

The intervertebral disc (IVD) is the main joint connecting two adjacent vertebral bones in the spine. It is composed of three closely connected parts: nucleus pulposus (NP), annulus fibrous (AF), and cartilage endplate (EP). In the process of IVDD, the decrease in proteoglycan and collagen in the extracellular matrix (ECM) directly reduces the hydration capacity of IVD, leading to the decrease in water content in NP, which in turn leads to intervertebral disc collapse and decreased disc height [7, 8]. Pfirrmann et al. [9] proposed the Pfirrmann classification of lumbar disc degeneration based on the characteristics of NP water content and disc height reflected in magnetic resonance imaging (MRI) T2WI images, which has been widely used in clinical practice. Studies have defined grade 4 and grade 5 discs (black discs) in Pfirrmann's grading system as degenerative discs, while those with grade 3 and below are defined as nondegenerative discs (white discs) [5, 10]. The cause of IVDD is not well known. In addition to age, IVDD is associated with obesity [11], smoking [12], and diabetes mellitus [10]. Genetic factors have also been shown to be associated with IVDD [13, 14]. Exploring the mechanism of IVD degeneration is helpful for the prevention and treatment of LBP, reducing social and economic burden and improving life quality.

IVDD is accompanied by molecular expressional changes which have the potential to serve as IVD degeneration markers, such as the downregulation of keratin 19 and N-cadherin [15, 16]. Increased inflammatory factors have also been found in degenerated IVDs [17]. Previous research reported that degenerated human IVDs are in a chronic inflammatory state [18, 19]. Studies have found increased expression of several proinflammatory factors in human degenerative IVDs such as interleukin-1 beta (IL-1*β*) and tumor necrosis factor alpha (TNF-*α*) [20]. A number of studies have mimicked IVDD by adding proinflammatory factors in vitro and in vivo [21, 22]. Our previous study has found the upregulation of fibrosis markers [23, 24], the late stage of chronic inflammation, in degenerated IVDs. In the mouse and rabbit model, abnormal remodeling of the collagenous reticular tissue in the NP was observed [25, 26]. Overall, these evidences suggest degenerative IVDs in a chronic inflammatory environment.

Cyclooxygenase-2 (COX2) is an “inducible” isoform of COX enzymes. Unlike COX1, COX2 expression is usually minimal, but when activated COX2 regulates prostaglandin E2 (PGE2) production which is involved in neuronal, metabolic, and immune system function, COX2 is involved in inflammation and is a crucial mediator of pain conduction [27]. COX2 has been shown to be regulated by, or regulates, many other inflammatory factors. Studies have demonstrated that treatment of rat serosal connective tissue mast cells with NGF induced COX2 [28]. IL-1*β* [29] treatment of human tendon cells and TNF-*α* treatment [30] of human lung fibroblasts both induced COX2 expression. IL-6 treatment of human NP cells induced PGE2 synthesis and COX2 expression [31]. Stimulation of COX2 also induced IL-8 production [32], suggesting that COX2 can further promote the inflammatory cascade. COX2 is also a critical pain mediator, and COX2-specific inhibitors have been used clinically for the treatment of painful conditions, including low back pain.

COX2 has been found to be induced in in vitro disc cell cultures by various degeneration inductors, such as TNF-*α* [33] and IL-1*β* [34]. However, up to date, the evidence on how COX2 expression changes in the natural process of IVD degeneration in human is rare. In this study, we verified the expression of COX2 in a relatively large scale of human specimens who visit the clinics due to low back pain. Here, we evaluated its expression by immunohistochemistry (IHC) and assessed its association with the degeneration grade of IVDs. We further analyzed the correlation of COX2 expression with the baseline information of the patients, as well as investigating the expression of COX2 in cultured human NP cells under the treatment with IL-1*β*, a well-accepted inflammatory mediator [35, 36] in IVDD. The aim is to gain a further understanding of COX2 in different degrees of IVDD, which can contribute to the understanding of IVDD pathogenesis and potential development of blocking strategies.

## 2. Materials and Methods

### 2.1. Participants

This study was conducted in the Orthopedics Department of the First Affiliated Hospital of the South China University of Technology between August 2019 and November 2020 with ethical approval from the Medical Ethical Committee from the South China University of Technology. 107 LBP participants undergoing spinal surgery after no response to conservative treatments for at least 6 weeks were included with informed patient consent. All patients received transdiscoscopic discectomy or lumbar fusion. IVD removed from these patients during surgery were collected as approved by the institutional review board (IRB). Among these, patients with spinal tumor and/or tuberculosis were excluded from this study. The enrolled patients had different degrees of low back pain. The degeneration grade of IVD was evaluated on a 1–5 scale according to the Pfirrmann classification system based on MRI T2WI [9]. Data about age, gender, body mass index (BMI), diabetes history, smoking history, and radiological imaging (MRI) were also recorded.

### 2.2. Immunohistochemistry

The expression of COX2 in the collected IVDs was analyzed by IHC. Tissue samples were fixed with 10% formalin and embedded in paraffin, cut into 5 *μ*M sections, and transferred to adhesive-treated slides. These slides were dried for 2 hours at 60°C, dewaxed for three times with xylene, and subjected to rehydration. After that, the slides were placed in an antigen repair apparatus (PT Module, Thermo Fisher Scientific) filled with antigenic repair solution (citric acid, pH = 6.0) in a microwave oven for antigenic repair. After heating at 100°C for 20 minutes and natural cooling, the slides were washed with PBS (pH 7.4) on a decolorization shaker for 3 times, 5 min each. 3% hydrogen peroxide was incubated for 25 min at room temperature (RT) to block the endogenous peroxidase activity. Then, the slides were blocked in 3% BSA for 30 min at RT. Afterwards, the sections were incubated overnight at 4°C with a primary rabbit antibody against COX2 (Abcam, ab15191) diluted in an antibody diluent (Servicebio, G2025) at the concentrations of 1 : 150 and 1 : 300, respectively. Then, the slides were incubated with a mouse anti-rabbit secondary antibody (Servicebio, GB23303) at the concentration of 1 : 200 at RT for 50 minutes and developed with diaminobenzidine (DAB) (Solarbio, DA1010), counterstained with hematoxylin (Servicebio, G1004), dehydrated in graded ethanol, and sealed with neutral balsam (Solarbio, 96949-21-2). Diagnostic scanners (3DHISTECH, Pannoramic MIDI) were used to randomly pick five microscopic images for each sample. The number of COX2-positive cells was manually counted, and the integrated optical density (IOD) and total cell number of each image were counted by using Image-Pro Plus (IPP6) software.

### 2.3. Degeneration Grading of the Clinical Samples

Two experienced trained staff independently graded the degeneration status of the patient IVDs based on sagittal MRI images (T2-weighted image (T2WI)) of the patient's spine according to the Pfirrmann grading system [9]. Images with conflicted judgments were evaluated again by working together until consensus was achieved for all patients.

### 2.4. Culture of Human Nucleus Pulposus Cells

The human nucleus pulposus cells used in this experiment were purchased from ScienCell. After defrosting, cells were inoculated into tissue culture dishes and cultured in DMEM complete medium supplemented with 1% penicillin-streptavidin, 1% L-glutamine, and 10% fetal calf serum in a 37°C humidified incubator. The cells were subcultured at a dilution of 1 : 3 when they reached 90% confluency. To test the induction of COX2 by IL-1*β*, cells at P3 were subjected to the addition of IL1*β* at the final concentration of 0, 5, 10, and 15 ng/ml for 24 hr. After 24 hr, the cells were harvested. RNA was isolated by using Trizol and assessed by using a Nanodrop bioanalyzer (Thermo Scientific, US). Reverse transcription of RNA to cDNA was done with an RNA to cDNA kit (Tsingke, PRC). Quantitative real-time PCR (qRT-PCR) of the expression of COX2 was performed on a StepOnePlus system (Applied Biosystems, Life technologies, US) using SYBR green real-time PCR master mixes (Tsingke, PRC). GAPDH was tested as an endogenous control. The relative quantification was achieved by the comparative CT method.

### 2.5. Statistical Evaluation

The normality of variables was assessed. For a comparison between two sets of data, data of normal distribution was expressed as mean ± standard deviation and the differences were evaluated by the *t*-test. Data with nonnormal distributions are represented by median (25th-75th percentile), and the differences were evaluated by the Mann–Whitney *U* test. For comparison among multiple groups of data, an ordinary one-way ANOVA test was used for comparison following normal distribution; the Kruskal-Wallis test was used for those who did not follow the normal distribution. The Spearman coefficient was used to assess the correlation between COX2 expression and the baseline information in the IVDD samples. Significance was set at *P* < 0.05. All statistical analyses were performed with SPSS 23.0 software (IBM, Chicago, USA).

## 3. Results

### 3.1. Age Is Associated with IVDD in Population Baseline Information

According to the grading system, we graded the disc samples collected from 107 patients. We have 7, 38, 57, and 5 cases of intervertebral discs at grades 2, 3, 4, and 5, respectively. The MRI images of the patients classified into Pfirrmann grades II to V are represented in Supplementary Figure 1. Apart from dividing the specimens into grades 2~5, we further adopted the grouping methods mentioned by Teraguchi et al. [5], which grouped grade 4 and 5 discs (black discs) as degenerative discs, while those with grade 3 and below were grouped as nondegenerative discs (white discs).  Table 1 shows the characteristics of 107 patients, among whom 62 cases (57.9%) were degenerative (black) discs and 45 cases (42.1%) were nondegenerative (white) discs. The population information, including gender, age, body mass index (BMI), diabetes mellitus (DM) history, and smoking history, was compared between the patients with black discs and those with white discs. Patients with black discs were significantly older (*P* < 0.001) and tend to have higher prevalence of DM (*P* = 0.051) than their counterparts though not significant difference was found. This is consistent with the previous report [10]. Other than age and DM, the other population baseline parameters were not statistically different in black discs compared to white ones.

### 3.2. Optimization of the IHC Staining of COX2 in Human Specimens

**Figure 1:**
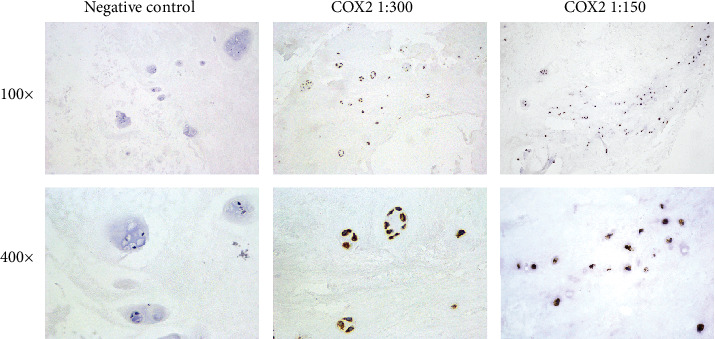
Illustration of the optimization of COX2 staining. IHC staining was performed with the COX2 antibody at 1 : 150 and 1 : 300 dilution. Negative control was obtained by omitting the primary antibody. Photos were taken at 100x and 400x magnification.

We tested the staining effect of the COX2 antibody at 1 : 150 and 1 : 300 dilution, as shown in Figure 1. Staining at both antibody concentrations can yield similar positive signals, while the staining at 1 : 300 dilution is slightly clearer than that at 1 : 150 dilution. Therefore, we adopted 1 : 300 dilution for the following experiments.

### 3.3. Differential Expression of COX2 in Disc NP at Different Degeneration Grades

**Figure 2:**
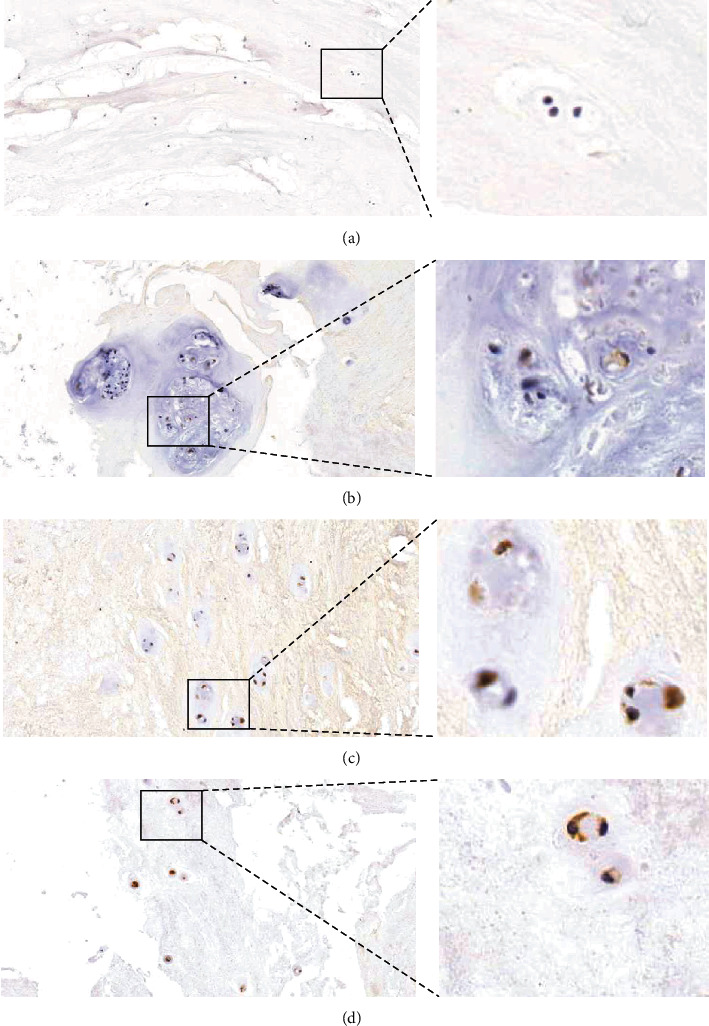
Immunohistochemical staining images of COX2 in human nucleus pulposus tissue. (a–d) The staining of NP at Pfirrmann grades II (a), III (b), IV (c), and V (d).

The staining results of COX2 in different degrees of IVDD are illustrated in Figure 2 and Supplementary Figure 2. The number of COX2-positive cells in grade 2 discs is low, which is increased in grade 3. In grade 4, the number of COX2-positive cells is significantly increased and the signal is enhanced. There is no visual difference in the number of COX2-positive cells and signal intensity between grade 5 and grade 4 NP.

### 3.4. Analysis of COX2 Expression in Human Discs at Pfirrmann Grades II to V

**Figure 3:**
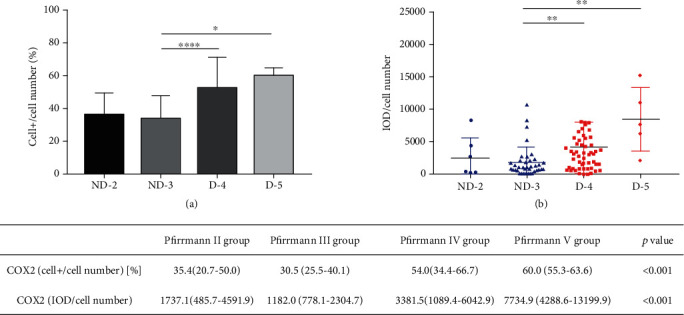
The expression of COX2 between grade 2, 3, 4, and 5 discs of the patients. (a) The percent of COX2+ cells in the total cell population in NP tissues at grades 2, 3, 4, and 5. (b) The IOD/cell number of COX2 in NP tissues at grades 2, 3, 4, and 5. ∗ represents *P* < 0.05, ∗∗ represents *P* < 0.01, ∗∗∗ represents *P* < 0.001, and ∗∗∗∗ represents *P* < 0.0001.

We use IPP6 software and two analyzing methods of IHC images to more accurately assess the expression of the target protein in human degenerative and nondegenerative NP tissues. The IOD/cell number represents the average COX2 signal intensity per cell, while the cell+/cell number represents the percentage of cells positive for COX2 expression. We conducted statistical analysis on the samples according to levels 2, 3, 4, and 5. The results are shown in Figure 3. COX2 positivity is highly significantly different between grades 3 and 4 (*P* < 0.0001) and grades 3 and 5 (*P* = 0.0213), respectively. COX2 expression density is highly significantly different between grades 3 and 4 (*P* < 0.01) and between grades 3 and 5 (*P* < 0.01), respectively. In summary, the expression levels of COX2 increase in high degenerative NP when compared to low/mild degenerative NP.

### 3.5. Analysis of COX2 Expression in Human Black and White Discs

**Figure 4:**
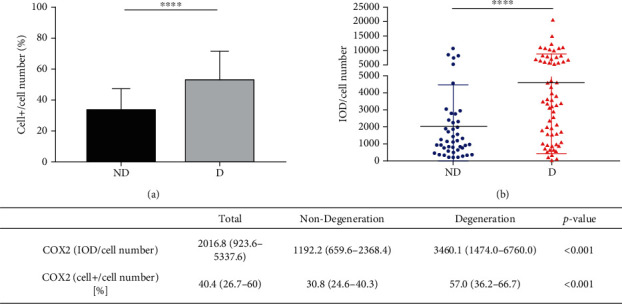
The expression of COX2 between human white and black discs of the patients. (a) The percent of COX2+ cells in total cell population between human degenerated and nondegenerated NP tissues. (b) The IOD/cell number of COX2 between human degenerated and nondegenerated NP tissues. D: degenerative (black) discs; ND: nondegenerative (white) discs. ∗∗∗ represents *P* < 0.001, and ∗∗∗∗ represents *P* < 0.0001.

Next, we grouped discs at grades 2 to 3 as white (nondegenerative) discs and discs at grades 4 to 5 as black (degenerative) discs and compared the expression of COX2 among them. The results are shown in Figure 4. For COX2 positivity, degenerative NP had significantly higher COX2+ cell proportion (57.9% vs. 30.8%, *P* < 0.001) than nondegenerative NP, respectively. For the cellular COX2 expression intensity, degenerative NP exhibits significantly higher IOD/cell number of COX2 (3460.1 vs. 1192.2, *P* < 0.001) than nondegenerative ones.

### 3.6. Spearman Analysis of Correlation between COX2 Expression and Baseline Information

The correlation between COX2 expression and the baseline information of the patients was assessed by the Spearman analysis. As shown in Table 2, the expression intensity of COX2, as represented by IOD/cell number, and the COX2 positivity, as represented by the cell+/cell number, are both significantly correlated with diabetes history (*P* = 0.031/*P* = 0.008). Body weight is significantly correlated with COX2 positivity, but not with COX2 expression intensity. Similarly, age is correlated with COX2 expression intensity but not with COX2 positivity. In summary, this indicates that COX2 expression is positively correlated with the occurrence of diabetes (Spearman correlation > 0), while it may have a correlation with age and weight.

### 3.7. IL-1*β* Stimulated COX2 Expression in Cultured Human NP Cells

**Figure 5:**
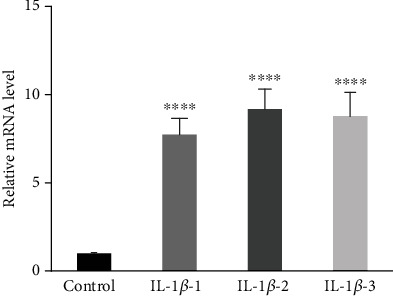
Expression of COX2 after treatment with IL-1*β* on human nucleus pulposus cells. The expressions of COX2 in each group were normalized to the expression of COX2 in the control group with no IL-1*β* treatment. IL-1*β*-1: 5 ng/ml, IL-1*β*-2: 10 ng/ml, and IL-1*β*-3: 15 ng/ml. COX2: cyclooxygenase 2; IL-1*β*: interleukin 1*β*. ∗∗∗∗ represents *P* < 0.0001.

IL-1*β* is known to induce inflammatory response in human or animal NP in the literature and has been widely utilized as an inflammation inducer in IVD in various studies [35, 36]. Here, we investigated whether the treatment of IL-1*β* may inflect the expression of COX2 in human NP cells in vitro. As shown in Figure 5, COX2 expression in human NP cells was all significantly upregulated by IL-1*β* at 5, 10, and 15 ng/ml within 24 hours. This result indicated that COX2 is involved in the inflammatory cascade induced by IL-1*β* in the NP.

## 4. Discussion

Not all IVD degeneration causes low back pain. While some degenerated IVDs cause discogenic LBP, some others are pain-free. A previous study has reported that none of the morphological changes, such as disc bulges, narrowing, Schmorl's nodes, or protrusions, can be distinguishable factors between asymptomatic and symptomatic patients [37, 38]. Inflammation is an important element of IVDD and might be the crucial factor distinguishing symptomatic and asymptomatic IVD degeneration [18].

COX2 is indicated in the inflammatory process in various tissues, such as Alzheimer's disease [39], Parkinson's disease [40], and diabetic kidney disease [41]. Its overexpression has been implicated as a biomarker for various types of cancers [42–44]. COX2 is also a crucial pain mediator. COX2 regulates the synthesis of prostaglandin E2 (PGE2), which plays an important role to induce radiculopathy. A COX2 selective inhibitor has been successfully developed as a commercially available drug to suppress pain. As a crucial mediator of pain and participant of inflammation signaling, COX2 may play an important role in IVDD and LBP development. Currently, the knowledge about the involvement of COX2 with IVDD is relatively low. A few studies have investigated the association of COX2 with IVDD in animal models and in vitro cell culture. In rat [45] and dog [46] models with induced IVD degeneration, increased expression of COX2 has been found in degenerated discs. In disc cell culture, IL-1*β* [34, 47] and TNF-*α* [48] have been found to elevate inflammatory gene expression including COX2. However, the information about COX2 expression in native human IVD at different degeneration grades is still scarce.

In this study, we looked into the baseline information of patients with different degrees of IVDD. We explored the association of COX2 with IVD degeneration with a relatively large scale of clinical human specimens. As a result, among all the baseline information investigated, none but age is shown to be associated with IVDD. The expression intensity of COX2 increased from grade 2 to grade 5, and the same trend is detected when comparing white discs to black discs. Consistently, cell positivity of COX2 also increases in more degenerative NP when compared to NP at lower degenerative grades. We further checked the expression of COX2 in cell culture in vitro and found that IL-1*β* treatment could upregulate COX2 expression in human NP cells, which is consistent with other findings [34, 47]. This indicates that COX2 is involved in the IL-1*β*-induced inflammatory process. In conclusion, the expression of COX2 is positively correlated with the degree of IVD degeneration and confirms the onset of inflammation in degenerated IVD. However, this study has its limitations in that it is an observational study to reveal the expressional changes of COX2 in different degeneration levels of human IVD only. We have not performed investigations of its expression in animal models of IVDD, which will be beneficial for consolidating the association of COX2 with IVDD.

The incidence of IVDD is high and age-dependent. A study showed that the prevalence rates of IVDD in the whole spine are 71% in men and 77% in women at age 50-, while the rates are over 90% at age 50+ both in men and women [5]. A large population-based study in South China showed that 40% of people under 30 suffer from lumbar disc degeneration, but the proportion in the 60+ group is approximately 100% [49]. In recent years, a young population showed an increased incidence rate. Makino et al. [50] reported that 31% of people under 20 at their first MRI examination were found to have IVDD. A recent review has evaluated the occurrence of IVDD ranging from kindergarten- to middle school-aged children and shows that LBP is rare in preschoolers and then increases until it becomes similar to that of adults at age 18 [51]. In our study, it is found that the age of patients in the IVDD group is significantly higher than that in the non-IVDD group, which is consistent with a number of previous reports. We did not find any association of BMI or smoking with IVDD in this study. Since the pathogenesis of the type I and type II diabetes is different, we further looked into the patient information and found that all the 5 patients involved in this study have type II diabetes. We did not find association of type II diabetes with IVDD in these patients. The possible reason is that the sample sizes of patients with diabetes or smoking history (5 each) in this study are too small to study the association of diabetes or smoking with IVDD. It is the same for our study that though COX2 expression is found to be positively correlated with diabetes through the Spearman analysis, the scientific significance of their association is undermined by the small sample size of diabetes patients in this study. Body weight has a correlation with COX2 cellular positivity, while age has an association with COX2 expression intensity, which indicates that COX2 expression may be connected with the body weight and age of the patients.

We realize that though the sample size was relatively large, due to the difficulty in obtaining healthy IVD samples, only degenerative IVDs at grades 2 to 5 of the Pfirrmann grading system were obtained. To solve this, apart from directly comparing the discs at different degeneration grades, we also adopted the criteria by Teraguchi et al. [5] to have the white disc group and black disc group to allow multiple examination of their association with IVDD. Also, this is only an observational study on COX2 expression in IVDD. Though we showed that IL-1*β* can regulate COX2 expression in the NP, much detail is lacking to reveal the upstream and downstream signaling of COX2 in IVDD. Further investigation on their roles in IVD inflammation and degeneration would help to understand the pathogenesis of IVDD in more detail and facilitate the development of repairing strategies.

In addition, the recent identification of endogenous progenitor cells with mesenchymal stem cell-like properties [52] in the IVD [53] brings forward a question of how IVD progenitor cells are involved in or respond to IVD inflammation. Further investigation on the association of IVD progenitor cells with IVD inflammation would be desirable.

## 5. Conclusion

The expression of COX2 increased with the degree of IVD degeneration, which highlights COX2 as a biomarker for IVD degeneration. Furthermore, IL-1*β* regulates COX2 expression in the NP, which indicates the possible involvement of inflammation and pain signaling in the process of IVD degeneration. Further investigation into the function of COX2 during IVDD is required to reveal its role in IVDD.

## Figures and Tables

**Table 1:** Population baseline between human degenerated and nondegenerated discs of the study patients.

	Total	Nondegeneration	Degeneration	*P* value
Patients (*n*)	107 [100]	45 [42.1]	62 [57.9]	—
Gender (*n* [%])	107 [100]	45 [100]	62 [100]	
Male	70 [65.4]	34 [75.6]	36 [58.1]	0.06
Female	37 [34.6]	11 [24.4]	26 [41.9]	
Age (years)	43.0 ± 14.9	35.8 ± 13.3	48.3 ± 13.9	<0.001
BMI	23.4 ± 3.4	23.1 ± 3.9	23.6 ± 3.1	0.485
Height (m)	1.66 ± 0.08	1.67 ± 0.07	1.66 ± 0.09	0.363
Weight (kg)	65 (56-70)	63 (55.8-71.5)	65 (57-70)	0.75
Diabetes (yes vs. no)	107 [100]	45 [100]	62 [100]	
Yes	5 [4.7]	0 [0]	5 [8.1]	0.051
No	102 [95.3]	45 [100]	57 [91.9]	
History of smoking (yes vs. no)	107 [100]	45 [100]	62 [100]	
Yes	5 [4.7]	2 [4.4]	3 [4.8]	0.924
No	102 [95.3]	43 [95.6]	59 [95.2]	

^∗^Values are expressed as mean ± standard deviation or number.

**Table 2:** Spearman analysis of correlation between COX2 and baseline information.

	COX2 positivity (cell+/cell number)		COX2 intensity (IOD/cell number)	
	Spearman correlation	*P* value	Spearman correlation	*P* value
Gender (*n* [%])	0.068	0.488	0.054	0.58
Age (years)	0.177	0.069	0.313	0.001
BMI	0.14	0.15	0.123	0.206
Height (m)	0.077	0.433	0.018	0.851
Weight (kg)	0.193	0.047	0.131	0.178
Diabetes history (yes or no)	0.209	0.031	0.254	0.008
Smoking history (yes or no)	-0.079	0.419	0.049	0.618

## Data Availability

The data used to support the findings of this study are available from the corresponding author upon request.

## Supplementary Materials

Supplementary Materials Supplementary Figure 1: MRI images of human intervertebral discs of Pfirrmann grades II to V. The red arrow indicates the harvested IVD level for the Pfirrmann classification. Supplementary Figure 2: representative COX2 immunohistochemical staining results of several human nucleus pulposus specimens at Pfirrmann grades II (line 1), III (line 2), IV (line 3), and V (line 4).
