# Supplementary material for: The Upregulation of COX2 in Human Degenerated Nucleus Pulposus: The Association of Inflammation with Intervertebral Disc Degeneration
Source: Mediators Inflamm. 2021 Oct 18;2021:2933199. doi: 10.1155/2021/2933199 (PMC8545564; doi:10.1155/2021/2933199)

Supplementary Figure 1. MRI images of human intervertebral discs of Pfirrmann grade II to V.

The red arrow indicates the harvested IVD level for Pfirrmann classification.

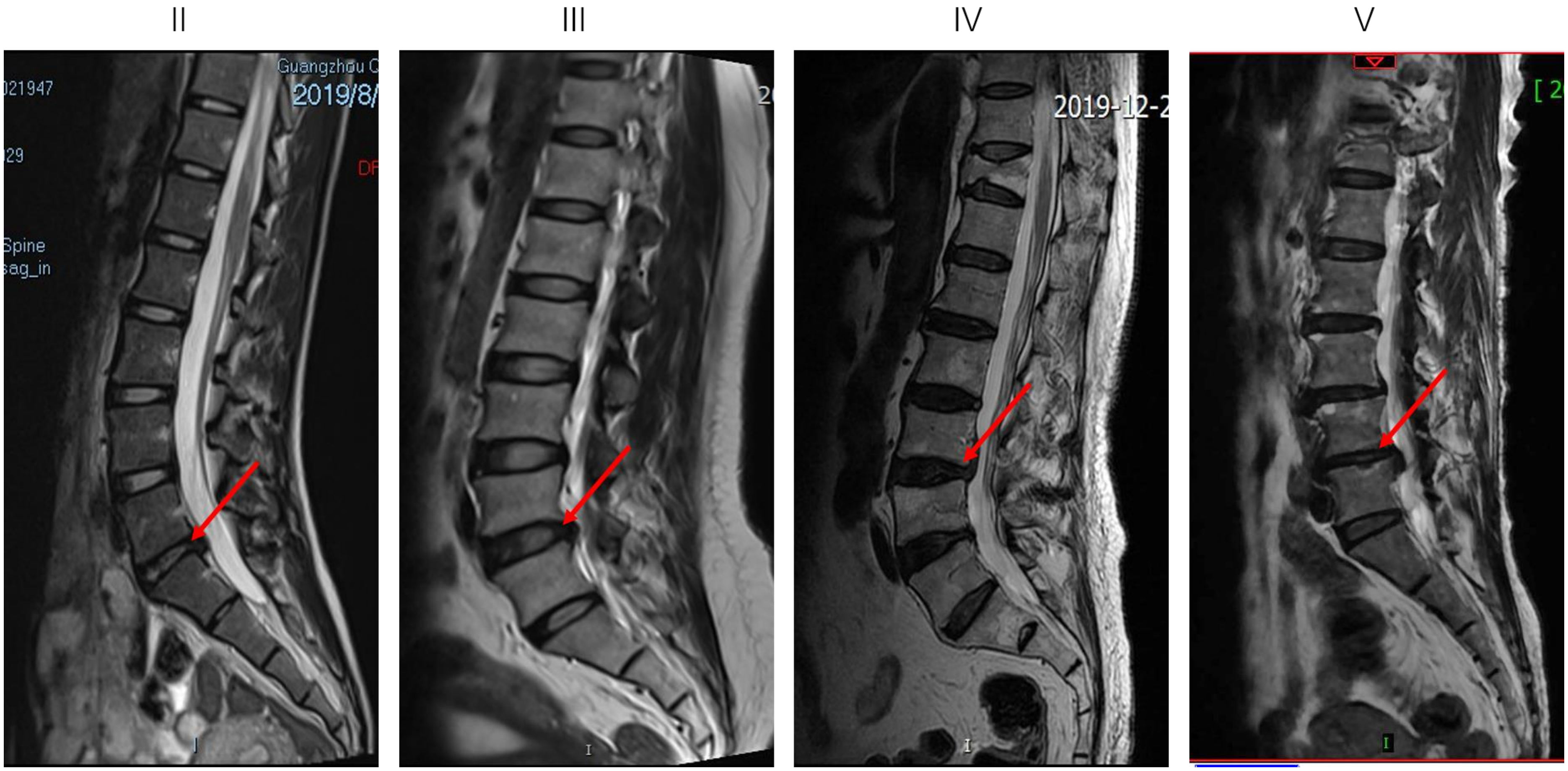

Supplementary Figure 2. Representative COX2 immunohistochemical staining results of several human nucleus pulposus specimens at Pfirrmann grade II (line 1), III (line 2), IV (line 3) and V (line 4).

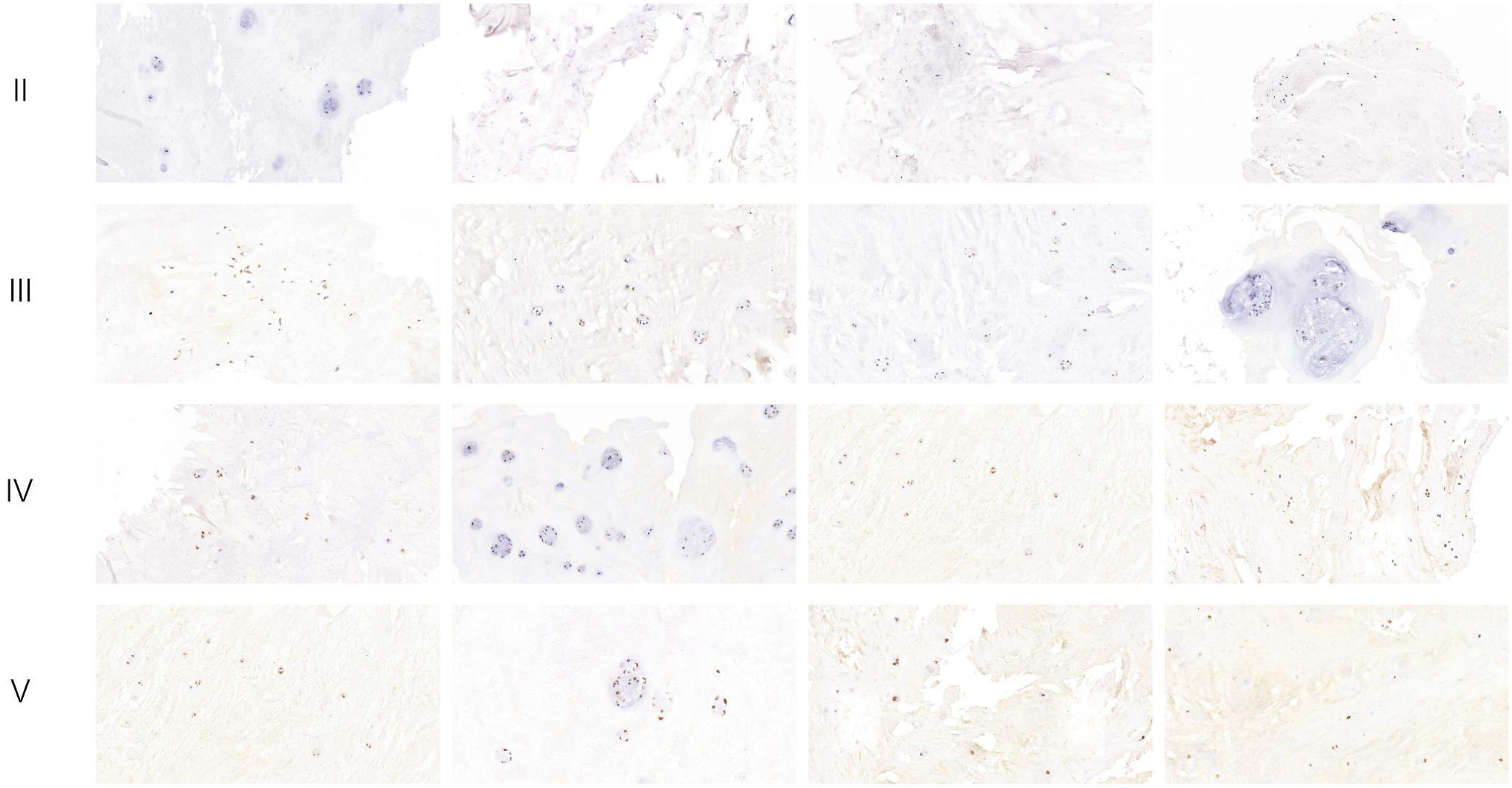

Supplement: Supplementary Materials — Supplementary Figure 1: MRI images of human intervertebral discs of Pfirrmann grades II to V. The red arrow indicates the harvested IVD level for the Pfirrmann classification. Supplementary Figure 2: representative COX2 immunohistochemical staining results of several human nucleus pulposus specimens at Pfirrmann grades II (line 1), III (line 2), IV (line 3), and V (line 4). [file 2933199.f1.zip › Supplementary File.pdf]
