# Supplementary figures and images for: The Upregulation of COX2 in Human Degenerated Nucleus Pulposus: The Association of Inflammation with Intervertebral Disc Degeneration
Source: Mediators Inflamm. 2021 Oct 18;2021:2933199. doi: 10.1155/2021/2933199 (PMC8545564; doi:10.1155/2021/2933199)

## Slide 1
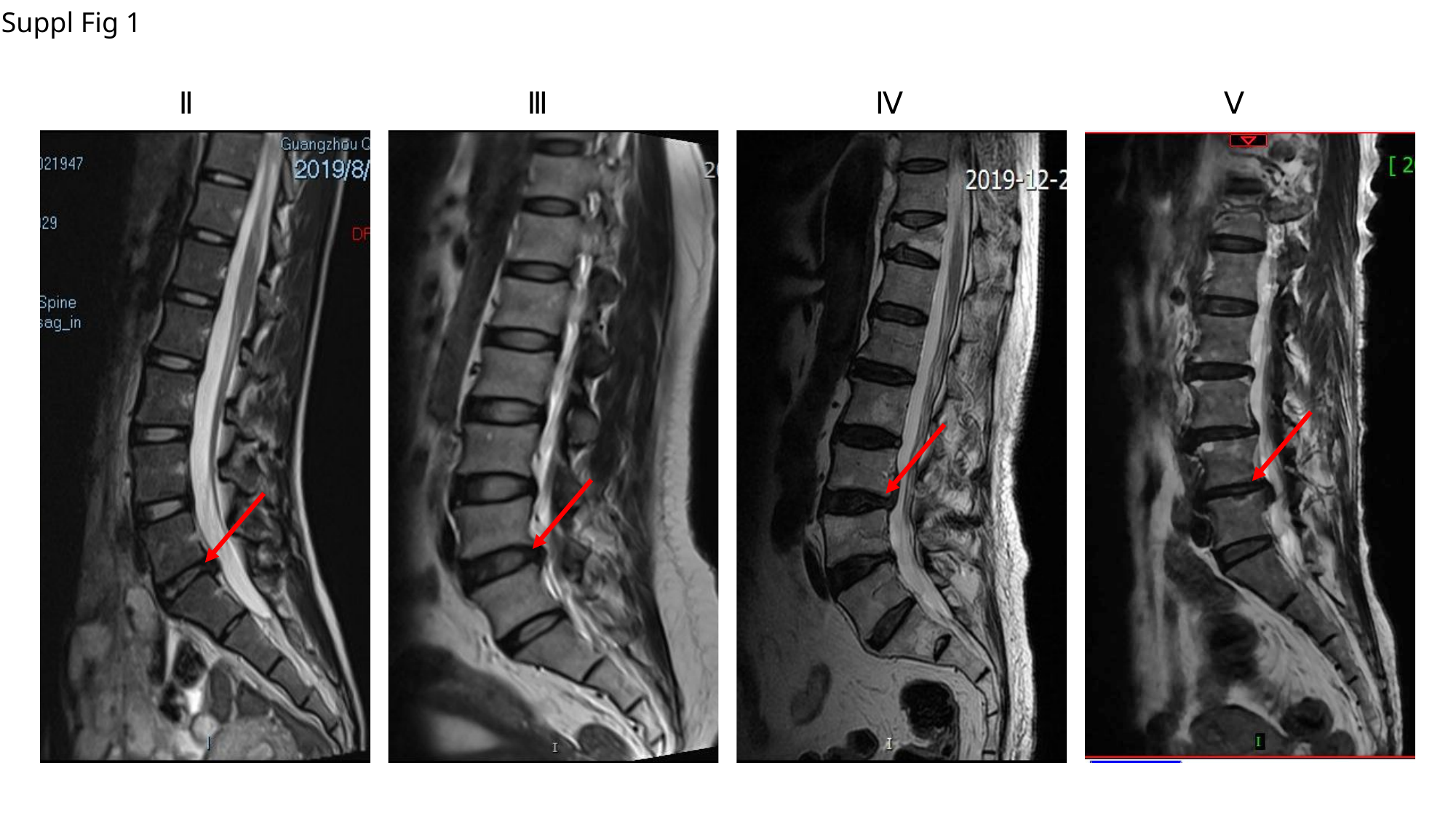

Suppl Fig 1
Ⅱ
Ⅲ
Ⅳ
Ⅴ

Supplement: Supplementary Materials — Supplementary Figure 1: MRI images of human intervertebral discs of Pfirrmann grades II to V. The red arrow indicates the harvested IVD level for the Pfirrmann classification. Supplementary Figure 2: representative COX2 immunohistochemical staining results of several human nucleus pulposus specimens at Pfirrmann grades II (line 1), III (line 2), IV (line 3), and V (line 4). [file 2933199.f1.zip › Suppl.Figure 1.pptx]

## Slide 1
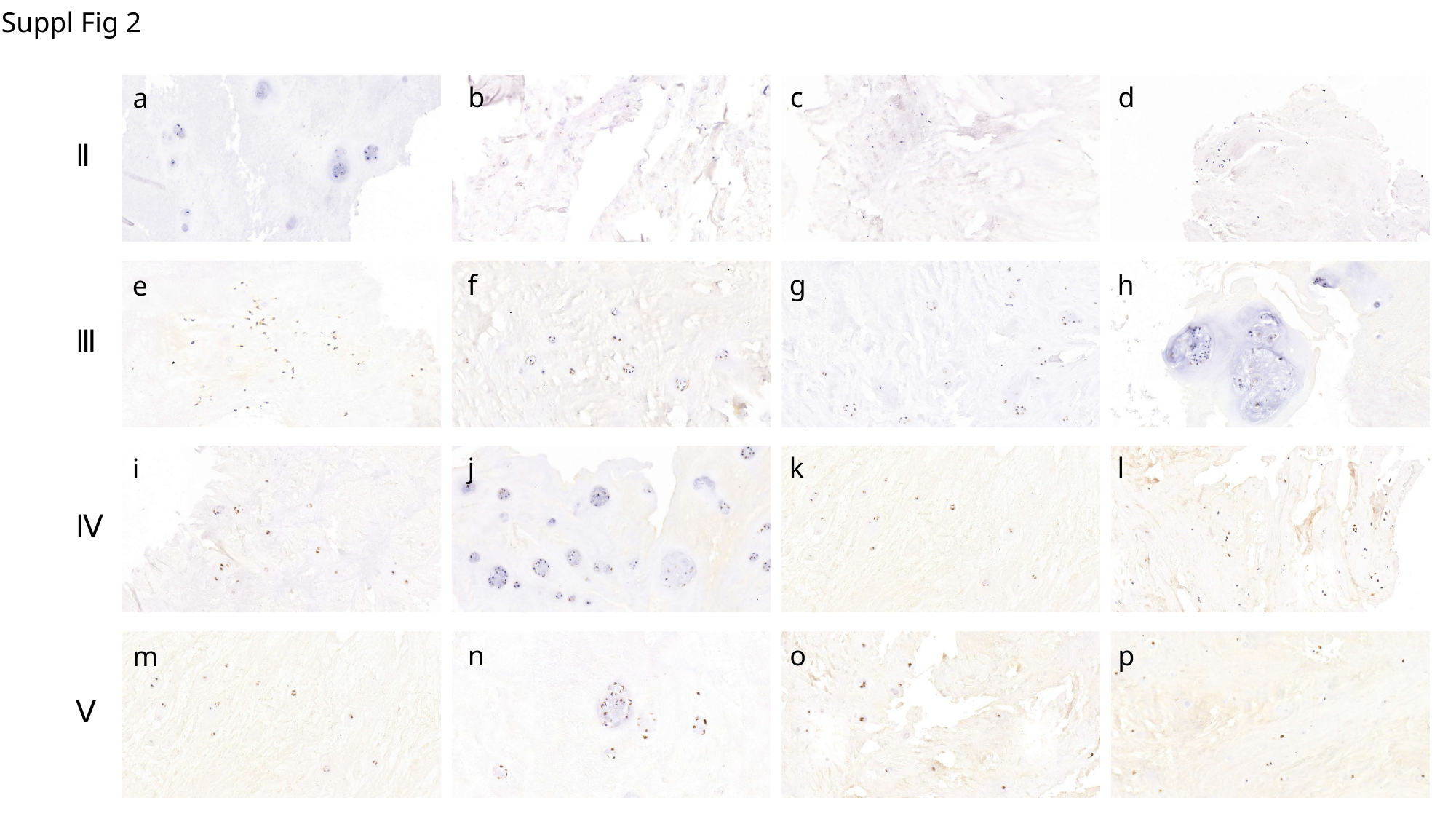

Suppl Fig 2
b
c
d
Ⅱ
Ⅲ
Ⅳ
Ⅴ
a
f
g
h
e
j
k
l
i
n
o
p
m

Supplement: Supplementary Materials — Supplementary Figure 1: MRI images of human intervertebral discs of Pfirrmann grades II to V. The red arrow indicates the harvested IVD level for the Pfirrmann classification. Supplementary Figure 2: representative COX2 immunohistochemical staining results of several human nucleus pulposus specimens at Pfirrmann grades II (line 1), III (line 2), IV (line 3), and V (line 4). [file 2933199.f1.zip › Suppl.Figure 2.pptx]
